# The relationship between intra-specific variation in the *Populus* transcriptome, stomatal development, and the metabolome in response to drought

**DOI:** 10.1186/1753-6561-5-S7-O32

**Published:** 2011-09-13

**Authors:** Erin T Hamanishi, Sherosha Raj, Olivia Wilkins, Barb R Thomas, Shawn D Mansfield, Aine L Plant, Malcolm M Campbell

**Affiliations:** 1 Faculty of Forestry, University of Toronto, 33 Willcocks Street, Toronto, Ontario M5S 3B3 Canada; 2 Department of Cell & Systems Biology, University of Toronto, 25 Willcocks St., Toronto, ON M5S 3B2 Canada; 3 Alberta-Pacific Forest Industries Inc. P.O. Box 8000 Boyle, Alberta, T0A 0M0, Canada, 5Department of Renewable Resources, University of Alberta, 731 General Services Building, Edmonton, AB T6G 2H1, Canada; 4 Department of Wood Science, University of British Columbia, 4030-2424 Main Mall, Vancouver, BC V6T 1Z4, Canada; 5 Department of Biological Sciences, Simon Fraser University, 8888 University Drive, Burnaby, BC V5A 1S6, Canada; 6 Centre for the Analysis of Genome Evolution and Function; Department of Cell & Systems Biology, University of Toronto, 25 Willcocks St., Toronto, ON M5S 3B2, Canada

## 

Drought is one of the most significant factors limiting tree growth. Trees in the genus *Populus* are particularly noted for their drought sensitivity; therefore, understanding the mechanisms by which these economically and ecologically important forest trees respond to drought is of paramount importance. The ability of *Populus* trees to contend with drought is dependent on the responsiveness of the genome, and in turn, the ability of the transcriptome to appropriately remodel growth, metabolism and development. Amassing evidence indicates that different species of *Populus* have divergent mechanisms and adaptations to contend with drought stress; however, individuals within a given species also display divergent drought responses. In order to investigate the intra-specific variation underpinning the drought response, we examined six genotypes of *P. balsamifera*. Using Affymetrix Poplar GeneChips, we found a positive correlation between the magnitude of drought-induced changes in the transcriptome and the capacity of the genotype to maintain growth. Surprisingly identifiable differences at the transcriptome were observed, and similar responses were observed within the metabolome. Although common drought responses could be identified within the species, the complexities of these responses must be taken into consideration when defining species- or genus-level drought responses.

